# E4-Ubiquitin ligase Ufd2 stabilizes Yap8 and modulates arsenic stress responses independent of the U-box motif

**DOI:** 10.1242/bio.010405

**Published:** 2015-08-14

**Authors:** Rita T. Ferreira, Regina A. Menezes, Claudina Rodrigues-Pousada

**Affiliations:** Instituto de Tecnologia Química e Biológica António Xavier, Universidade Nova de Lisboa, Av. da República, EAN, Oeiras 2781-901, Portugal

**Keywords:** Ufd2, U-box domain, AP-1 like transcription factor Yap8, Arsenic

## Abstract

Adaptation of *Saccharomyces cerevisiae* cells to arsenic stress is mediated through the activation of arsenic detoxification machinery by the Yap8 transcription factor. Yap8 is targeted by the ubiquitin proteasome system for degradation under physiological conditions, yet it escapes proteolysis in arsenic-injured cells by a mechanism that remains to be elucidated. Here, we show that Ufd2, an E4-Ubiquitin (Ub) ligase, is upregulated by arsenic compounds both at mRNA and protein levels. Under these conditions, Ufd2 interacts with Yap8 mediating its stabilization, thereby controlling expression of *ACR3* and capacity of cells to adapt to arsenic injury. We also show that Ufd2 U-box domain, which is associated to the ubiquitination activity of specific ubiquitin ligases, is dispensable for Yap8 stability and has no role in cell tolerance to arsenic stress. Thus, our data disclose a novel Ufd2 role beyond degradation. This finding is further supported by genetic analyses showing that proteins belonging to Ufd2 proteolytic pathways, namely Ubc4, Rad23 and Dsk2, mediate Yap8 degradation.

## INTRODUCTION

Arsenic (As) is a toxic element widely spread in nature due to natural and anthropogenic sources (Mandal and Suzuki, 2002). Several studies have revealed that As mediates toxicity via inducing the production of reactive oxygen species (ROS), inhibiting DNA repair, altering DNA methylation and increasing cell apoptosis (Flora, 2011; Jomova et al., 2011). Paradoxically, due to its anticancer effect, arsenic trioxide (As_2_O_3_) has been proposed as a front-line agent for treatment of acute promyelocytic leukemia (APL) (Mathews et al., 2011; Iland and Seymour, 2013).

In the yeast *Saccharomyces cerevisiae*, protective response to arsenic stress involves multiple cellular mechanisms. They include the activation of antioxidant defense machinery, the calcium-signaling pathways, the repression of the high affinity iron uptake system, the adjustment of sulfur metabolism to enhance GSH biosynthesis associated to intra- and extra-cellular chelation of arsenic, the regulation of cell cycle progression as well as enhancement of proteasomal degradation of misfolded/damaged proteins (Menezes et al., 2008; Migdal et al., 2008; Ferreira et al., 2012; Jacobson et al., 2012; Thorsen et al., 2012; Batista-Nascimento et al., 2013). The most important adaptive mechanism triggered in cells exposed to As(V) and As(III) requires the activity of Yap8 transcription factor. It modulates As detoxification by the activation of *ACR2* and *ACR3* genes encoding an arsenate reductase, which catalyses the conversion of As(V) into As(III), and an arsenite efflux pump, respectively (Wysocki et al., 1997, 2004; Bobrowicz and Ulaszewski, 1998; Haugen et al., 2004; Rodrigues-Pousada et al., 2004). Yap8 is the most divergent member of the yeast AP-1 like family of transcription factors displaying restricted DNA-binding specificities in comparison with the other family members (Ilina et al., 2008; Amaral et al., 2013). Its activity is tightly controlled at different levels. Previously, we have reported that Yap8 shifts between the cytoplasm and the nucleus under non-stressed conditions, while arsenic compounds trigger its retention in the nucleus (Menezes et al., 2004). A further mechanism of Yap8 regulation relies on the post-translational control of its protein levels by the ubiquitin-proteasome pathway (UPP). It was shown that Yap8 is ubiquitinated and degraded by the proteasome under physiological conditions, and upon arsenic injury, it escapes degradation by a mechanism that is not yet elucidated (Di and Tamas, 2007).

The cycle of ubiquitin (Ub) attachment to the lysine residues of target proteins commonly involves the catalytic activities of E1-Ub-activating and E2-Ub-conjugating enzymes, and E3-Ub-ligases (Finley, 2009; Hochstrasser, 2009; Ciechanover and Stanhill, 2014). Nevertheless, efficient multiubiquitination of specific substrates also requires the activity of E4 enzymes, which work in association with E1s, E2s and E3s to catalyse Ub chain assembly necessary for recognition and degradation by the 26S proteasome (Hoppe, 2005). Among the few yeast E4 enzymes, the ubiquitin fusion degradation enzyme Ufd2 is the best characterized and the first identified member (Koegl et al., 1999). It belongs to a family of proteins containing a domain with 70 amino acids at their C-terminus, termed the U-box, conserved among eukaryotes (Hatakeyama and Nakayama, 2003). This domain is associated to the elongation of Ub chains being structurally related to the RING finger domain found in certain E3-Ub ligases (Aravind and Koonin, 2000; Tu et al., 2007). The U-box is generally considered as the essential functional unit of E4s, however, it was reported that the U-box of human UFD2a is not required for the proteasomal turnover of p73 (Hosoda et al., 2005).

The mechanisms by which Yap8 circumvents proteolysis under As stress are still elusive constituting a matter of investigation in the present study. Although Ufd2 was shown to act as an E4 enzyme active in degradation, our study leads to novel insights on Ufd2 function in yeast. *UFD2* deletion in the yeast genome reveals that Yap8 is destabilized under As conditions, its transcriptional activity is decreased, and cellular tolerance to As compounds is compromised. Interestingly, Ufd2 function in Yap8 regulation seems to be independent of the U-box domain. Our results describe the involvement of Ufd2 in a new function beyond proteolysis.

## RESULTS

### Yap8 levels are tightly controlled by arsenic

**Fig. 1.**
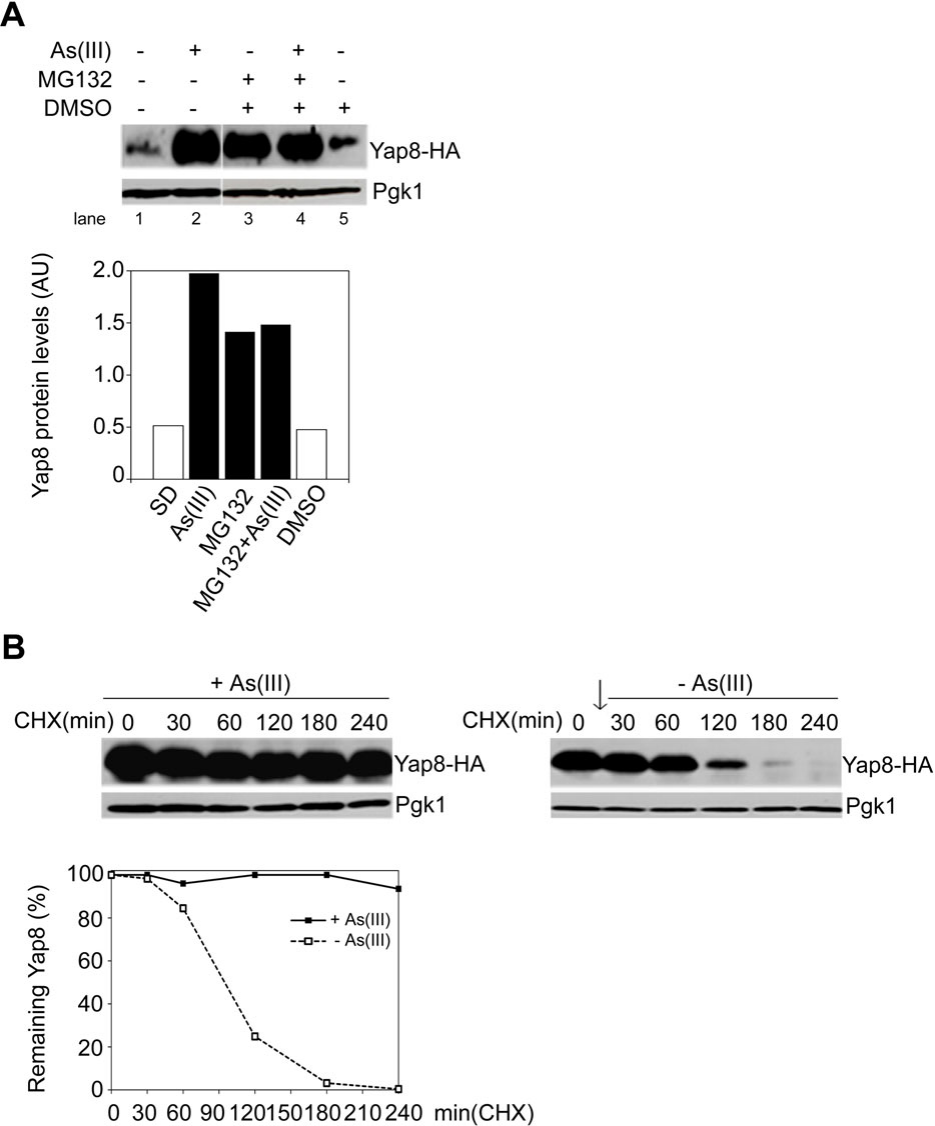
**Arsenic triggers Yap8 stabilization circumventing proteasomal degradation.** (A) Wild type cells exposed to arsenite exhibit enhanced Yap8 levels. BY4742 wild type (WT) cells expressing Yap8-HA were left untreated (SD) or exposed to 1.5 mM As(III) alone or in combination with 100 µM MG132 and DMSO, for 90 min. The graph represents relative Yap8 levels normalized against Pgk1 levels (arbitrary units, AU). (B) As(III) triggers Yap8 stabilization. Cells were first exposed to 1.5 mM As(III) for 30 min, cycloheximide (CHX) was then added to a final concentration of 0.1 mg/ml up to 240 min and protein extracts were subjected to immunoblotting using a anti-HA antibody. Pgk1 was used as loading control. The graph represents the percentage of remaining Yap8 after CHX addition. Left panel: As(III) was maintained in the medium; estimated Yap8 half-life >240 min. Right panel: As(III) was removed from the medium before CHX addition; estimated Yap8 half-life is 94 min. The arrow indicates the removal of As(III). Representative experiments are shown.

Arsenic stress responses in *S. cerevisiae* require the AP-1 like transcription factor Yap8, which drives the expression of genes involved in As detoxification processes (Menezes et al., 2004). Aiming at elucidating further the mechanisms underlying Yap8 regulation, we analysed by immunoblotting the levels and the stability of HA-tagged Yap8 in wild type (WT) cells, either in the presence or the absence of arsenite (Fig. 1). Yap8-HA levels were shown to be low in cells incubated under control conditions, and increased in response to 90 min treatment with As(III) (Fig. 1A, lanes 1 and 2). Furthermore, inhibition of proteasome activity with MG132 led to an increase of Yap8-HA levels under non-inducing conditions (Fig. 1A, lanes 1 and 3), but not in the presence of As(III), as indicated by cell co-treatment with MG132 and As(III) (Fig. 1A, lanes 2 and 4). These data indicate that Yap8 is not degraded by proteasome under arsenic stress conditions. In DMSO-treated cells, Yap8 levels are comparable to those observed in the control condition (Fig. 1A, lanes 1 and 5). We have then determined Yap8 stability in the presence and absence of As by using the protein synthesis inhibitor cycloheximide (CHX). For that, cells were first pre-treated with 1.5 mM arsenite for 30 min followed by CHX treatment up to 240 min. It is clear that Yap8 levels were enhanced during this period (Fig. 1B, left panel) in contrast to what does occur if As was completely removed from the medium before CHX treatment (Fig. 1B, right panel). Measurement of Yap8 half-life in both conditions revealed that it is strongly reduced in the absence of As (>240 min vs 94 min).

These findings are in agreement with those previously reported using different As conditions (Di and Tamas, 2007), and are consistent with the notion that Yap8 is a substrate for proteasomal degradation under physiological conditions whereas it is stabilized by arsenic.

### The E4-Ufd2 enzyme interacts with Yap8 under arsenic stress conditions

**Fig. 2.**
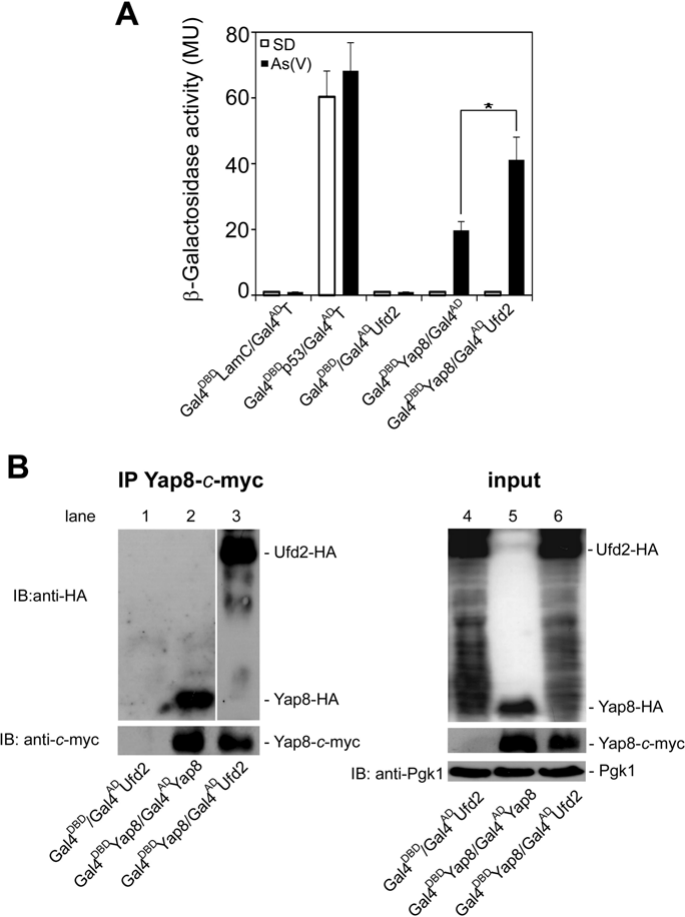
**Yap8 interacts with Ufd2 upon arsenic stress.** (A) Yeast two-hybrid assays reveal Ufd2 as a Yap8-interaction partner. The Y187 strain was co-transformed with plasmids encoding *GAL4^DBD^Lam/GAL4^AD^T*, *GAL4^DBD^p53/GAL4^AD^T*, *GAL4^DBD^/GAL4^AD^UFD2*, *GAL4^DBD^YAP8*/*GAL4^AD^*or *GAL4^DBD^YAP8*/*GAL4^AD^UFD2* and β-galactosidase activity was measured in cells challenged or not with 2 mM As(V) (MU, Miller Units). Values represent the mean±standard deviation (s.d.) of three biological replicates and statistical differences denoted as *P<0.05. (B) Ufd2 co-immunoprecipitates together with Yap8. Y187 cells co-transformed with *GAL4^DBD^/GAL4^AD^UFD2* (lanes 1 and 4), *GAL4^DBD^YAP8/GAL4^AD^YAP8* (lanes 2 and 5) or *GAL4^DBD^YAP8*/*GAL4^AD^UFD2* (lanes 3 and 6) were exposed to 2 mM As(V) for 60 min and Gal4^DBD^Yap8, bearing a *c*-myc epitope, was immunoprecipitated with anti-*c*-myc antibody. Immunoblotting was performed using anti-HA, anti-*c-*myc and anti-Pgk1 antibodies. A representative experiment is shown. IP, immunoprecipitation; IB, immunoblotting.

To get further insights into the molecular basis of Yap8 stabilization that circumvents degradation upon arsenic stress, we have screened a yeast two-hybrid cDNA library fused to the Gal4 activation domain (Gal4^AD^), using Gal4 DNA binding domain-Yap8 (Gal4^DBD^Yap8) fusion as a bait protein. Yap8 is strongly activated by arsenic, therefore, to increase the likelihood of identifying new Yap8-interaction partners, the cDNA library was generated from cells induced with a sub-lethal dose of pentavalent inorganic arsenic As(V). Performing the screening in the presence of 0.5 mM As(V) allowed us to identify new Yap8-interaction partners (data not shown), among them the ubiquitin fusion degradation enzyme Ufd2. In order to assess the specific interaction between Yap8 and Ufd2, cells co-expressing Gal4^DBD^Yap8 and Gal4^AD^Ufd2 along with the respective controls, were treated or not with 2 mM As(V) for 60 min, and interaction was followed through induction of *lacZ* reporter gene in quantitative β-galactosidase assays (Fig. 2A). A high β-galactosidase activity was detected in Gal4^DBD^Yap8/Gal4^AD^Ufd2-expressing cells only under conditions where Yap8 is activated, i.e. in the presence of arsenic. The β-galactosidase signal observed in control cells expressing Gal4^DBD^Yap8/Gal4^AD^ and challenged with arsenic is due to the Yap8 transactivation potential (Menezes et al., 2004). Notwithstanding, Gal4^DBD^Yap8/Gal4^AD^Ufd2 interaction has yielded β-galactosidase activity values significantly higher than those determined for Gal4^DBD^Yap8/Gal4^AD^. As a positive control we have used cells expressing the well-known interacting proteins, p53 and SV40 T-antigen, fused to the Gal4^DBD^ and Gal4^AD^, respectively (Gal4^DBD^p53/Gal4^AD^T) (Li and Fields, 1993). Similarly to what we observed for non-stressed cells co-expressing Gal4^DBD^Yap8/Gal4^AD^Ufd2, we did not detect β-galactosidase activity for Gal4^DBD^/Gal4^AD^Ufd2 and Gal4^DBD^LamC/Gal4^AD^T control cells.

The specificity of Yap8/Ufd2 interaction was further corroborated by co-immunoprecipitation using Gal4^DBD^Yap8 and Gal4^AD^Ufd2 constructs, which also comprise the *c*-myc and HA epitopes, respectively, and similar conditions to that of the two-hybrid analysis (Fig. 2B). The fusion Yap8-*c-*myc was efficiently immunoprecipitated using the anti-*c-*myc antibody (Fig. 2B, lanes 2 and 3). Co-immunoprecipitation signals were only detected in cells co-expressing Yap8-*c*-myc together with Ufd2-HA (Fig. 2B, lane 3) revealing the specific interaction between Yap8 and Ufd2. The formation of Yap8 homodimeric complexes (R.A.M., unpublished observations; Di and Tamas, 2007) was used as positive control (Fig. 2B, lane 2). As revealed by immunoblotting analysis of whole cell extracts, Yap8 and Ufd2 were properly expressed in all conditions tested (Fig. 2B, lanes 4-6). To avoid artefacts that could interfere with Yap8 and Ufd2 interaction, including the presence of Gal4 tags and overexpression of fusion proteins, reciprocal co-immunoprecipitation assays were performed using BY4742 cells carrying *YAP8-c-myc* and *UFD2-HA* in the centromeric (*CEN-ARS*) vectors and under the control of native promoters. Yap8-*c*-myc was efficiently co-immunoprecipitated together with Ufd2-HA in cells challenged with As(III) for 90 min but not in cells left untreated (supplementary material Fig. S1).

Overall, these data reveal Yap8 as an interaction partner of Ufd2 in cells exposed to arsenic stress conditions.

### Ufd2 modulates arsenic stress responses

**Fig. 3.**
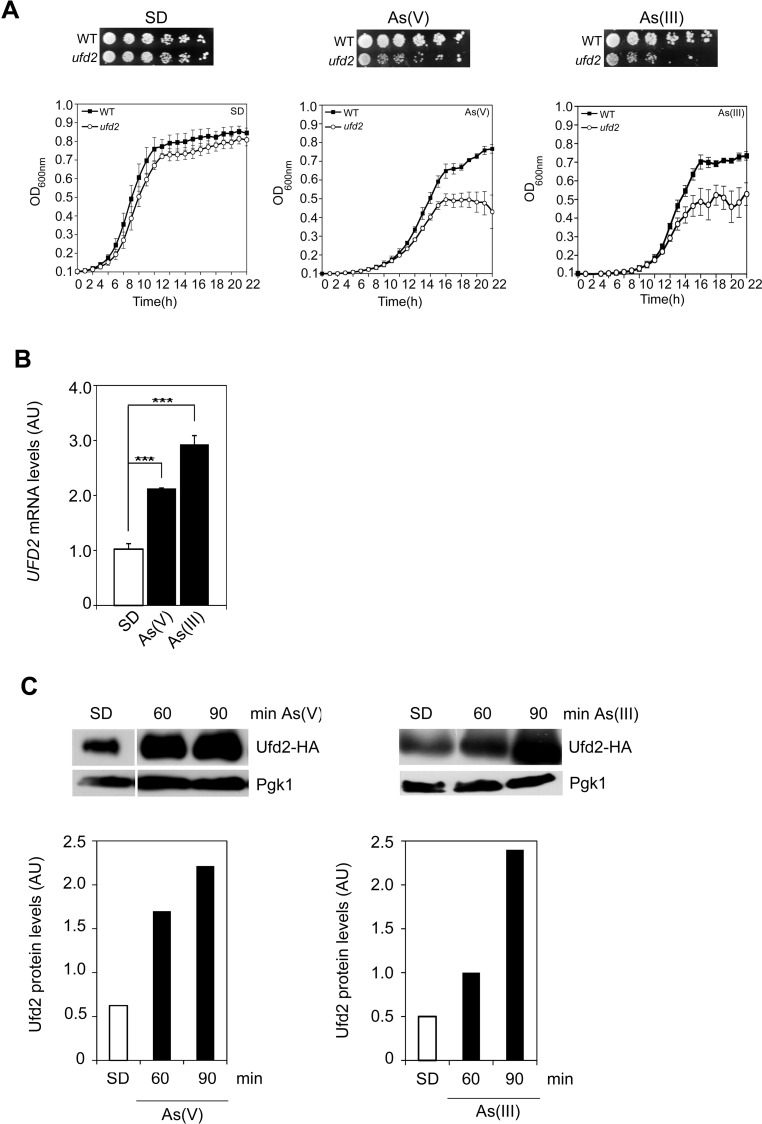
**Ufd2 mediates arsenic tolerance.** (A) *ufd2* cells are sensitive to arsenic stress. Exponential phase BY4742 wild type (WT) and the *ufd2* mutant were serially diluted and spotted onto SC media supplemented or not with 1.5 mM As(III) or 2 mM As(V) or 1.5 mM As(III). SD, control. Growth was recorded after 2 days incubation at 30°C. A representative experiment is shown. Cell growth was also monitored by means of growth curves. Exponential phase BY4742 WT and *ufd2* mutant cells were exposed or not to 2 mM As(V) or 1.5 mM As(III) for 22 h and OD_600_ was monitored in intervals of 1 h. The curves represent the mean±s.d. of three biological replicates. (B) *UFD2* is induced in cells injured with arsenic. BY4742 cells were challenged or not with 1.5 mM As(III) or 2 mM As(V) or 1.5 mM As(III) and *UFD2* mRNA levels were determined by qRT-PCR (AU, Arbitrary Units). Values represent the mean±s.d. of three biological replicates and statistical differences denoted as ***P<0.001. (C) Ufd2 protein levels increase during arsenic stress. BY4742 cells expressing Ufd2-HA were treated with 2 mM As(V) or 1.5 mM As(III) and harvested at the indicated time-points. SD, control. Immunoblottings were performed using anti-HA and anti-Pgk1 antibodies. Pgk1 was used as loading control. The graphs represent relative Ufd2 levels (AU, Arbitrary Units). Representative experiments are shown.

The results above suggest that Ufd2 may play a role in cells exposed to arsenic stress. To characterize the mechanisms by which Ufd2 is implicated in arsenic stress responses, we first evaluated its requirement to arsenic tolerance. We have therefore carried out phenotypic growth assays as well as growth curves of the WT and isogenic *ufd2* mutant strains, either in the absence or presence of both As(V) or As(III). *UFD2* gene is not essential for yeast cell viability (Bohm et al., 2011), however its deletion leads to a slight growth impairment in the control condition (Fig. 3A), as previously reported (Yoshikawa et al., 2011; Marek and Korona, 2013). Remarkably, *ufd2* displayed sensitivity to both As(V) and As(III) stresses (Fig. 3A), which is restored after the reintroduction of an episomal copy of *UFD2* in the mutant strain (supplementary material Fig. S2). In agreement with the notion that As(III) is more toxic than As(V) (Ratnaike, 2003), we also noted that *UFD2* deletion severely impairs cell growth in the presence of As(III). These results bring to light a novel role for Ufd2 in yeast arsenic adaptation.

We have further examined how As stress stimuli affect Ufd2. First, it was analysed arsenic-mediated changes in *UFD2* gene expression by qRT-PCR. As shown in Fig. 3B, *UFD2* mRNA levels increased 2-fold and 3-fold after 90 min exposure to As(V) and As(III), respectively. Consistent with *UFD2* mRNA analyses, Ufd2 protein levels were also enhanced upon As(V) or As(III) stress (Fig. 3C).

Altogether, these results show the activation of Ufd2 by arsenic suggesting that it is a novel regulator of metalloid stress response. Since Ufd2 function seems to be more critical in cells exposed to As(III), this condition was chosen for further studies.

### Ufd2 regulates Yap8 stabilization

**Fig. 4.**
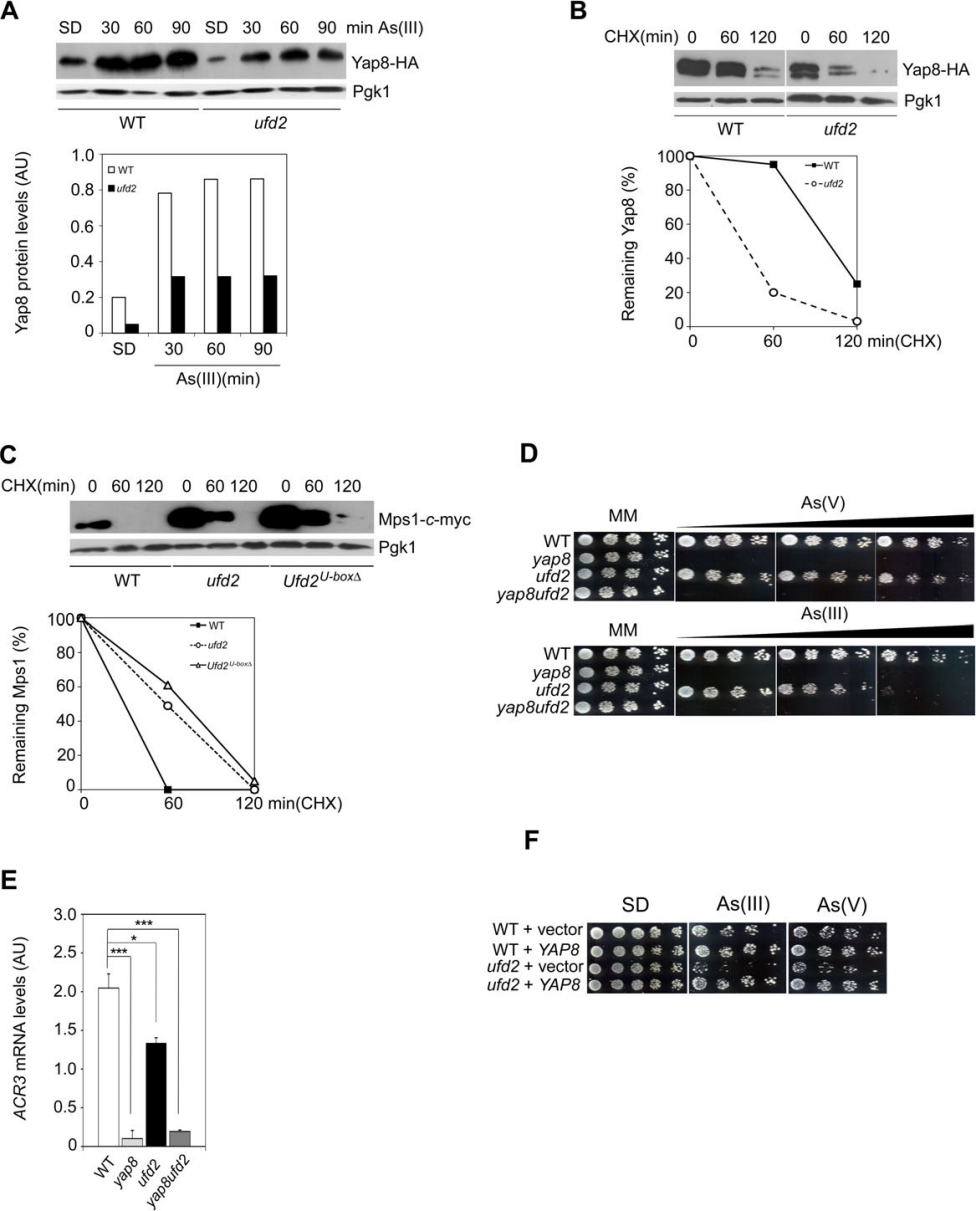
**Ufd2 mediates Yap8 stabilization.** (A) Yap8 levels are reduced in *ufd2* mutant cells compared to the wild type strain. BY4742 wild type (WT) and *ufd2* mutant strains expressing Yap8-HA were incubated with 1.5 mM As(III), harvested at the indicated time-points and subjected to immunoblotting using anti-HA and anti-Pgk1 antibodies. The graph represents relative Yap8 levels (AU, Arbitrary Units). (B) Yap8 is destabilized in the *ufd2* mutant. The same strains were first exposed to 1.5 mM As(III) for 90 min, washed and subsequently treated with 0.1 mg/ml cycloheximide (CHX) up to 120 min prior to immunoblotting with the antibodies indicated above. The graph represents the percentage of remaining Yap8 protein after CHX addition. Estimated Yap8 half-life is 98 min in the WT strain and 37 min in the *ufd2* mutant. (C) Mps1 stability is increased in *ufd2* and *Ufd2^U-boxΔ^* mutant cells in comparison to WT strain. BY4741 WT, *ufd2* and *Ufd2^U-boxΔ^* mutant strains carrying the *GAL1^promoter^MPS1-c-myc* construct were induced with galactose before being challenged with glucose and 0.1 mg/ml CHX. Cells were harvested at the indicated time-points and subjected to immunoblotting using anti-*c*-myc and anti-Pgk1 antibodies. The graph represents the percentage of remaining Mps1 protein after CHX addition. A representative experiment is shown. (D) Epistasis analyses of *YAP8* and *UFD2*. Exponential phase BY4742 WT, *yap8*, *ufd2* and *yap8ufd2* cells were serially diluted and spotted onto MM media supplemented or not with increasing concentrations of As(V) (up to 2 mM; upper panel) or As(III) (up to 1.5 mM; lower panel). Growth was recorded after 2 days incubation at 30°C. A representative experiment is shown. (E) *ACR3* expression is similar in the double *yap8ufd2* and single *yap8* mutants. The same strains referred in D were challenged with 1.5 mM As(III) for 90 min and *ACR3* mRNA levels were determined by qRT-PCR (AU, Arbitrary Units). Values represent the mean±s.d. of three biological replicates and statistical differences denoted as *P<0.05 or ***P<0.001. (F) *YAP8* overexpression recovers *ufd2* growth in cells exposed to arsenic stress. Exponential phase BY4742 WT and the *ufd2* mutant strain overexpressing *YAP8* or the vector alone were serially diluted and spotted onto SD media supplemented with 1.5 As(III) or 2 mM As(V). Growth was recorded after 2 days incubation at 30°C. A representative experiment is shown.

Ufd2 was shown to be involved in proteasomal degradation pathways (Liu et al., 2010). On the other hand, our data suggest that interaction of Ufd2 with Yap8 protects yeast cells against the toxicity of As compounds. To clarify the functional relationship between Ufd2 and Yap8, we have monitored the kinetics of Yap8 fused to HA epitope (Yap8-HA) induced by As(III) in WT and *ufd2* strains. As shown in Fig. 4A, *ufd2* displayed lower Yap8 levels when compared to WT cells indicating that Yap8 stability may be compromised in the absence of Ufd2.

We next examined Yap8 stability in WT and *ufd2* using CHX. Given that both Ufd2 and Yap8 are highly expressed in cells exposed to 1.5 mM arsenite for 90 min (Figs 3C and 4A), this condition was used to induce Yap8 expression in the cycloheximide chase experiments. After arsenic removal, cells were resuspended in fresh media containing CHX and incubated up to 120 min. Remarkably, Yap8 turnover rate was significantly increased in the *ufd2* mutant (half-life 37 min) as compared to the WT (half-life 98 min) (Fig. 4B). Moreover, we also observed a reduced Yap8 stability in *ufd2* cells exposed to 2 mM arsenate for 60 min prior to CHX treatment (supplementary material Fig. S3). Altogether, these results indicate that Ufd2 regulates Yap8 stabilization. To support the specificity of Ufd2-dependent Yap8 stabilization, it was monitored the Mps1 kinase turnover, a well known substrate of Ufd2 degradation pathway (Liu et al., 2011). We therefore performed CHX chase assays to assess the stability of *c*-myc tagged Mps1 under the regulation of *GAL1*-inducible promoter (supplementary material Table S2) in WT, *ufd2* and *Ufd2^U-boxΔ^* strains. The latter corresponds to a mutant in which the U-box domain, essential for Ufd2-mediated Mps1 proteolysis, was deleted (Liu et al., 2011). As expected, Mps1 was readily degraded in WT cells and the absence of Udf2 as well as of Udf2 U-box domain clearly decreases its degradation rates, consistent with Mps1 being targeted to proteasome (Fig. 4C).

Our data suggest that impaired Yap8 levels observed in *ufd2* cells may be responsible for their reduced tolerance to arsenite. Aiming at strengthening this hypothesis we have performed epistasis analyses to compare cellular growth and expression of the Yap8 target gene *ACR3* in WT, single *yap8* and *ufd2* mutants as well as in the double *yap8ufd2* mutant. In agreement with previous findings, *yap8* is very sensitive to arsenic stress due to a severe downregulation of *ACR3* expression as revealed by qRT-PCR (Fig. 4D,E) (Menezes et al., 2004; Wysocki et al., 2004). The growth patterns and *ACR3* expression profile of the double *yap8ufd2* mutant are almost identical to those observed for *yap8* (Fig. 4D,E) implying that Ufd2 seems to contribute to arsenic tolerance through the regulation of Yap8 stability and transcriptional activity (Fig. 4A,B,E). In contrast, the *ufd2* mutant is more resistant to arsenic than *yap8* being only sensitive to high doses of As(III) and As(V) (Figs 3A and 4D). In line with the notion that *ACR3* expression levels determine the extent of arsenite tolerance, *ACR3* mRNA levels were significantly higher in *ufd2* cells than in the *yap8* strain (Fig. 4E). Additionally, *YAP8* overexpression in the *ufd2* mutant restores arsenic tolerance of this strain (Fig. 4F) indicating that enhancement of *YAP8* expression compensates for lower protein stability in the absence of Ufd2.

Collectively, these results point out Ufd2-mediated stabilization as a further regulatory mechanism contributing to the tight control of Yap8 activity.

### Ufd2 U-box motif is not required for Yap8 stability and arsenic tolerance

**Fig. 5.**
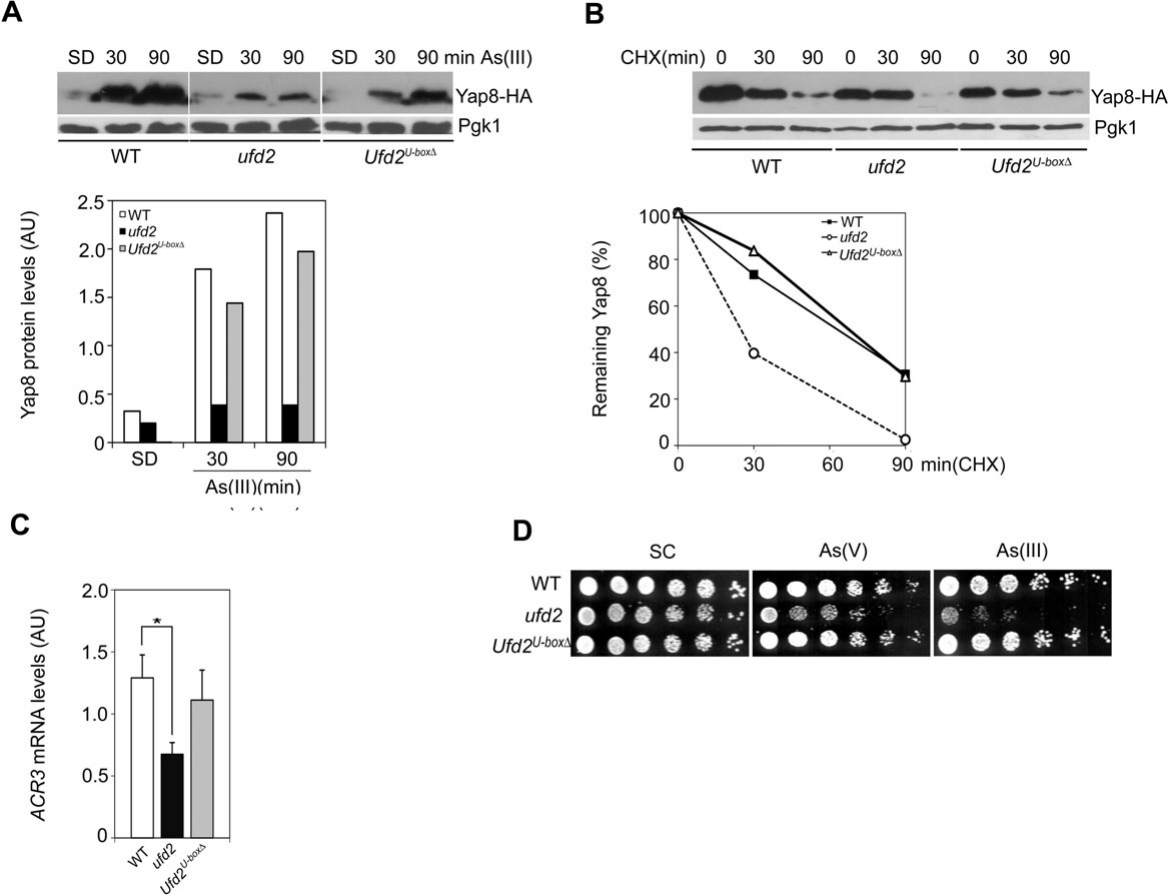
**Ufd2 U-box motif is not required for Yap8 stabilization.** (A) Yap8 levels are unaffected in the *Ufd2^U-boxΔ^* mutant strain compared to the wild type strain. BY4741 wild type (WT), *ufd2* and *Ufd2^U-boxΔ^* strains expressing Yap8-HA were incubated with 1.5 mM As(III), harvested at the indicated time-points and subjected to immunoblotting using anti-HA and anti-Pgk1 antibodies. The graph represents relative Yap8 levels (AU, Arbitrary Units). A representative experiment is shown; SD, control. (B) Yap8 stability is similar in WT and *Ufd2^U-boxΔ^* mutant strains. The same strains were first exposed to 1.5 mM As(III) for 90 min, washed and subsequently treated with 0.1 mg/ml cycloheximide (CHX) up to 90 min prior to immunoblotting, as indicated above. The graph represents the percentage of remaining Yap8 protein after CHX addition. Estimated Yap8 half-life is 63 min in the WT strain, 25 min in *ufd2* and 67 min in *Ufd2^U-boxΔ^*. (C) *ACR3* mRNA levels are unaltered in the *Ufd2^U-boxΔ^*. The same strains were challenged with 1.5 mM As(III) for 90 min and *ACR3* mRNA levels were determined by qRT-PCR (AU, Arbitrary Units). Values represent the mean±s.d. of three biological replicates and statistical differences denoted as *P<0.05. (D) The *Ufd2^U-boxΔ^* mutant is tolerant to arsenic stress. Exponential phase BY4741 WT, *ufd2* and *Ufd2^U-boxΔ^* cells were serially diluted and spotted onto SC media supplemented or not with 2 mM As(V) or 1.5 mM As(III). Growth was recorded after 2 days incubation at 30°C. A representative experiment is shown.

Several reports indicate that Ufd2 U-box motif is essential for its ubiquitination activity (Aravind and Koonin, 2000; Tu et al., 2007). We have therefore investigated whether this activity was also required for Yap8 stabilization and cell tolerance to arsenic stress. BY4741 isogenic strains were used for these experiments since the *Ufd2^U-boxΔ^* mutant is available exclusively in this yeast background (Liu et al., 2011). First, it was examined Yap8-HA kinetics in WT, *ufd2* and *Ufd2^U-boxΔ^* strains induced for 90 min with As(III). Corroborating the data from the BY4742 strain, Yap8 levels were found to be reduced in the *ufd2* mutant (Fig. 5A). Remarkably, the *Ufd2^U-boxΔ^* mutant displayed similar Yap8 levels as compared to the WT strain indicating that Ufd2 U-box domain is not involved in the regulation of Yap8 protein levels under arsenic stress. Next, Yap8 stability was monitored in the same strains. Cells were induced for 90 min with As(III) after which arsenic was removed and cells were treated with CHX for further 90 min. Supporting previous results obtained in the BY4742 strain (Fig. 4B), Yap8 stability is compromised in the *ufd2* mutant (Fig. 5B, half-life 25 min). Notwithstanding, *Ufd2^U-boxΔ^* mutant cells displayed similar Yap8 half-life compared to WT cells (67 and 63 min, respectively) confirming that Ufd2 U-box motif is dispensable for Yap8 stabilization during arsenic stress.

Having established that Ufd2 mediates Yap8 stabilization independent of the U-box motif, these strains were used to cement the premise that Yap8 stabilization correlates with the enhancement of Yap8 activity and tolerance to arsenite stress. We have then analysed *ACR3* expression in WT, *ufd2* and *Ufd2^U-boxΔ^* cells by qRT-PCR and found that the *Ufd2^U-boxΔ^* mutant and the WT strain exhibit similar *ACR3* mRNA steady state levels, which are higher than the *ufd2* mutant (Figs 4E and 5C). Consequently, cells lacking the U-box domain were tolerant to 1.5 mM As(III) and 2 mM As(V), as it was the WT strain, while growth of *ufd2* was impaired under these conditions (Figs 3A, 4D and 5D). In agreement with our previous results showing that Yap8 is not regulated at the transcriptional level (Menezes et al., 2004), *YAP8* mRNA levels were similar in WT, *ufd2* and *Ufd2^U-boxΔ^* cells (data not shown). These results indicate that Ufd2 does not regulate *YAP8* expression, yet it is involved in post-translational mechanisms modulating Yap8 levels.

Altogether, these data firmly establish that the U-box domain is not essential for Ufd2 regulation of Yap8 levels and further support the hypothesis that arsenic-sensitive phenotype of the *ufd2* mutant is mediated via diminished Yap8 activity.

### Ufd2 stabilizes Yap8 independent of the ubiquitin-proteasome pathway

**Fig. 6.**
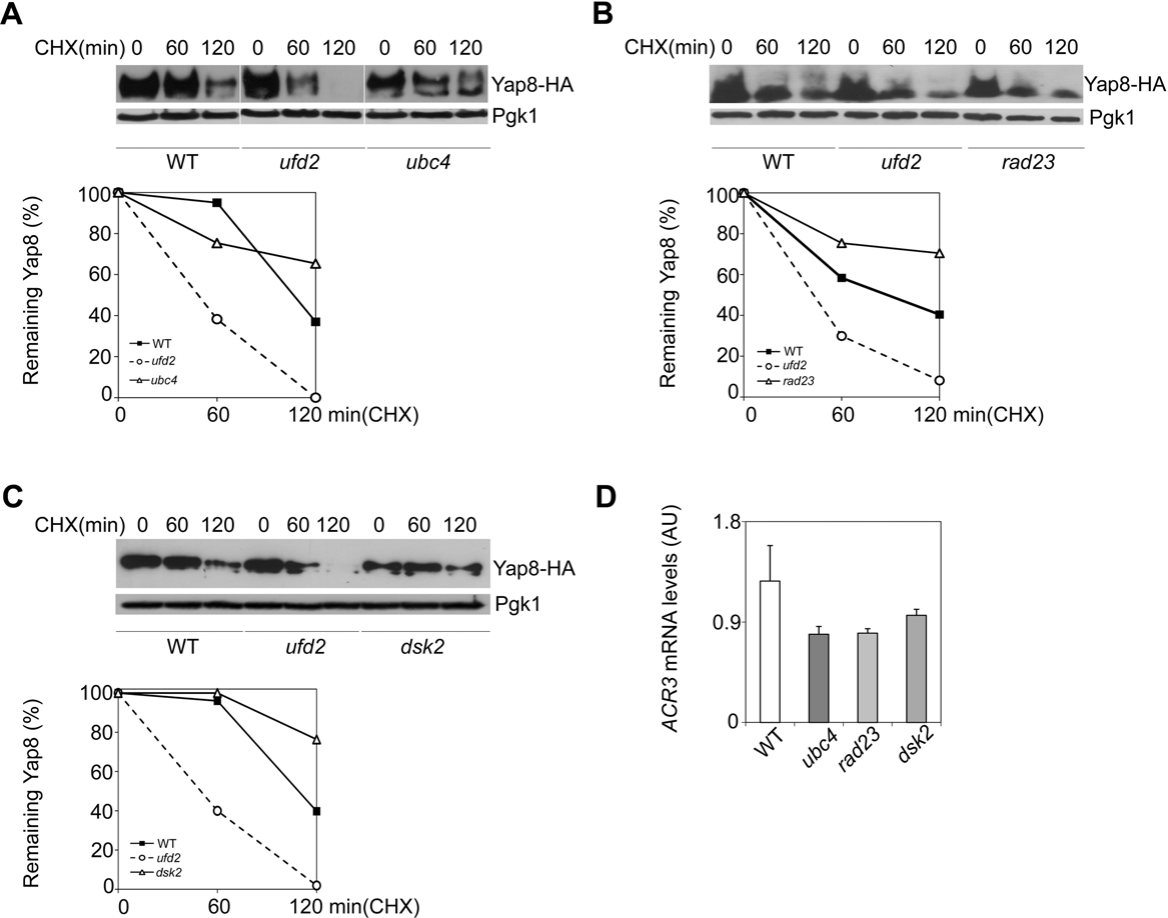
**Ubiquitin proteasome pathway (UPP) enzymes Ubc4, Rad23 and Dsk2 do not interfere with Yap8 stability in arsenic-exposed cells.** BY4742 wild type (WT), *ubc4* (A), *rad23* (B) and *dsk2* (C) mutant strains expressing Yap8-HA were first exposed to 1.5 mM As(III) for 90 min, washed and subsequently treated with 0.1 mg/ml cycloheximide (CHX) up to 120 min prior to immunoblotting using anti-HA and anti-Pgk1 antibodies. The graphs represent the percentage of remaining Yap8 protein after CHX addition. Representative experiments are shown. (D) *ACR3* mRNA levels remain unaltered in *ubc4*, *rad23* and *dsk2* mutant cells. The same strains were challenged with 1.5 mM As(III) for 90 min and *ACR3* mRNA levels were determined by qRT-PCR (AU, Arbitrary Units). Values represent the mean±s.d. of three biological replicates. No significant statistical differences were observed.

As to determine whether Ufd2 role on Yap8 stabilization is connected to UPP we next investigated the requirement of the UPP components Ubc4, Rad23 and Dsk2 to Yap8 stability under arsenic stress conditions. The E2 enzyme Ubc4 is upstream of Ufd2 in the ubiquitination process of proteins targeted to proteasome (Jentsch et al., 1990; Sommer and Seufert, 1992) whereas Rad23 and Dsk2 are Ufd2-downstream players bridging ubiquitinated proteins to the proteasome (Chen and Madura, 2002; Funakoshi et al., 2002; Kim et al., 2004). Importantly, the concerted action of Ufd2, Rad23 and Dsk2 in the degradation of Mps1 kinase was already described (Liu et al., 2011). In contrast to the reduced Yap8 stability observed in the *ufd2* strain, Yap8 half-life was found to be higher than the WT strain in the *ubc4, rad23* and *dsk2* mutants challenged with arsenite (Fig. 6A-C). Furthermore, arsenite-mediated upregulation of *ACR3* is not significantly affected in these mutants (Fig. 6D) indicating that Ubc4, Rad23 and Dsk2 are dispensable for Ufd2-mediated Yap8 stabilization and transcriptional activity. These results are also consistent with the observation that Yap8 is stabilized in mutants with defective proteasomal activity (Di and Tamas, 2007).

Overall, these data disclose a novel Ufd2 role beyond degradation independent of Ubc4, Rad23 and Dsk2.

## DISCUSSION

The b-ZIP transcription factor Yap8 plays a key role in arsenic stress responses as it regulates expression of the arsenic detoxification genes *ACR2* and *ACR3*. It was previously shown that Yap8 activity is controlled at different levels, including its degradation by the UPP under physiological conditions and stabilization upon arsenic stress (Fig. 1) (Menezes et al., 2004; Di and Tamas, 2007). Nevertheless, the mechanisms by which Yap8 circumvents degradation under arsenic stress have not yet been deciphered. Here, we move a step forward in the characterization of these mechanisms by identifying the E4-Ub fusion degradation enzyme Ufd2 as a Yap8-interaction partner (Fig. 2) and a mediator of arsenic tolerance in *S. cerevisiae.* This conclusion is supported by data indicating that *ufd2* cells are less tolerant to either arsenite or arsenate than the parental WT strain (Figs 3A, 4D and 5D) and that *UFD2* expression is highly induced in cells challenged with these compounds (Fig. 3B,C).

The mechanism by which Ufd2/Yap8 interaction governs yeast adaptation to arsenic stress was unveiled by the demonstration that *ufd2* cells exhibit decreased Yap8 levels as a consequence of impaired protein stability (Figs 4A,B and 5A,B). Hence, the reduction of Yap8 levels in the *ufd2* mutant compromises *ACR3* expression thereby affecting yeast tolerance to arsenic stress (Figs 3A; 4D,E and 5C,D). The molecular pathway underlying Ufd2 role in cellular protection against arsenic injury was strengthened by epistasis analyses revealing that *yap8* and the double *yap8ufd2* mutant cells challenged with arsenic compounds display almost identical growth patterns and *ACR3* expression profiles (Fig. 4D,E). These data, reinforced by the demonstration that Ufd2 and Yap8 interact *in vivo*, clearly connect Ufd2-mediated arsenic tolerance to Yap8 stability and transcriptional activity (Fig. 4A,B,E). The fact that *YAP8* overexpression restores arsenic tolerance of *ufd2* cells (Fig. 4F) may indicate that Ufd2 operates an additional regulatory mechanism contributing to the tight control of Yap8 activity.

The recapitulation of Ufd2-dependent degradation of Mps1, an essential protein kinase required for spindle pole body (SPB) duplication (Liu et al., 2011), was performed in our experimental conditions to corroborate the specificity of Ufd2-mediated Yap8 stabilization (Fig. 4C). Thus, maintenance of Yap8 levels by the E4-Ub ligase Ufd2, a well-known component of the proteolytic machinery, represents the most striking finding of this work and the first report of a yeast Ub-ligase stabilizing a transcription factor. Interestingly, it was shown that the Ufd2 human orthologue, UFD2a, also attenuates degradation of the ΔNp63α regulator by increasing its half-life and transcriptional activity (Chatterjee et al., 2008). The current knowledge of Ub ligases is far more complex and diverse. For example, the E3 MDM2 mediates p53 regulation via the UPP (Moll and Petrenko, 2003), but it can also bind to the p53 relative protein, p73, stabilizing its levels (Ongkeko et al., 1999).

Yeast Ufd2 contains a U-box domain, which is present in proteins from yeast to humans, and is associated to the enzymatic activity of E4s necessary for their proteolytic function (Tu et al., 2007). To show that Ufd2 stabilization role upon Yap8 is independent of its proteolytic function, various assays using the *Ufd2^U-boxΔ^* mutant were performed. The data resumed in Fig. 5 show that the U-box motif is required neither for Yap8 stability nor its activity. In line with the hypothesis that Ufd2 mediates arsenic tolerance through the regulation of Yap8 levels, the U-box domain is dispensable for cell adaptation to arsenic injury. This novel Ufd2 function was further reinforced by genetic analyses showing that Ubc4, Rad23 and Dsk2, components of proteolytic pathways, are not required for Yap8 stabilization (Fig. 6). Taking together, these results indicate that Ufd2, usually associated to protein degradation pathways, may be a bi-functional protein exerting also a role beyond proteolysis regulation.

To conclude, we have shown here a novel function of the Ub-fusion degradation enzyme Ufd2 in the regulation of Yap8 stability and consequently arsenic tolerance. However, further efforts are required for a deep understanding of the molecular events regulating Yap8 stability by Ufd2.

## MATERIALS AND METHODS

### Strains, plasmids and growth conditions

The yeast strains and plasmids used in this study are listed in supplementary material Tables S1 and S2, respectively. To generate the *yap8ufd2* double mutant, the *UFD2* gene was disrupted in the *yap8* mutant strain using the oligonucleotides 1 and 2 (supplementary material Table S3) and the microhomology PCR method (Gueldener et al., 2002). *UFD2* deletions were confirmed by PCR using genomic DNA and *UFD2* specific oligonucleotides (supplementary material Table S3, oligonucleotides 3 and 4). HA-tagged *UFD2* was generated by homologous recombination into the pRS416 vector (Agilent Technologies, Santa Clara, CA, USA) previously linearized with *Sma*I (Fermentas™ Thermo Fisher Scientific Inc., Rockford, IL, USA), using the In-Fusion^®^ HD Cloning Plus CE kit (Clontech Laboratories, Inc., Mountain View, CA, USA) and oligonucleotides indicated in supplementary material Table S3 (oligonucleotides 10 to 13) to generate pRS416-*UFD2-HA*. *UFD2-HA* was then sub-cloned into YCplac111 vector (Agilent Technologies) as a *Sma*I fragment. *GAL4^AD^UFD2* fusion was obtained by PCR amplification of *UFD2* gene lacking the ATG codon (oligonucleotides 8 and 9, supplementary material Table S3) and subsequent cloning into the pGADT7-Rec vector (Clontech Laboratories, Inc.), previously linearized with *Sma*I. Sequence integrity of *UFD2* constructions was confirmed by sequencing with the oligonucleotides listed in supplementary material Table S3 (oligonucleotides 5 to 7). PCR reactions were performed using the Phusion High-Fidelity DNA Polymerase (New England Biolabs, Ipswich, MA, US) and a Trio-ThermoBlock (BioMetra, Goettingen, Germany).

Yeast strains were maintained in YPD solid medium [1% yeast extract, 2% bactopeptone, 2% glucose and 2% of agar (Difco™ Thermo Scientific Inc.)]. They were grown in synthetic complete (SC) medium [0.67% ammonium sulfate-yeast nitrogen base without amino acids (Difco™ Thermo Scientific Inc.), 2% glucose, supplemented with essential amino acids and bases], synthetic defined (SD) medium [0.67% ammonium sulfate-yeast nitrogen base without amino acids, 2% glucose, supplemented with required amino acids and bases] or minimal medium (MM) [0.67% ammonium sulfate-yeast nitrogen base without amino acids, 2% glucose, supplemented only with amino acids and bases corresponding to the respective auxotrophic markers] at 30°C, with orbital agitation (200 rpm). Absorbance at 600 nm (OD_600)_ was measured using a SmartSpec™ 3000 (Bio-Rad Laboratories, Hercules, CA, USA). When indicated, 1.5 mM sodium arsenite [As(III), NaAsO_2_], 2 mM sodium arsenate [As(V), Na_2_HAsO_4_.7H_2_O], 100 µM MG132 or 0.1 mg/ml cycloheximide (CHX), all purchased from Sigma-Aldrich (St. Louis, MO, USA), were added to cultures. Phenotypic growth assays were carried out by spotting 5 μl of sequentially diluted (∼5×10^3^ to 10 cells) early exponential phase cells (OD_600_ 0.5±0.05) onto the surface of the SC, SD or MM medium containing As(III) or As(V). Growth was recorded after 2 days at 30°C. For the growth curves, early exponential phase cultures were diluted to 0.1±0.01, and were exposed or not to As(III) for 24 h at 30°C, with agitation, in a 96-well microplate. OD_600_ was monitored in intervals of 1 h using the Epoch™ BioTek spectrophotometer (Winooski, VT, US). *MPS1-c-myc* encoding cells were pre-grown to early exponential phase (OD_600_ 0.5±0.05) in YEP-Raffinose (2% peptone, 1% yeast extract, 2% D-glucose, 1% raffinose) liquid media, resuspended in YEP-Galactose (2% peptone, 1% yeast extract, 2% galactose) liquid media and incubated for 180 min at 30°C with orbital agitation (200 rpm).

The bacterial *Escherichia coli* strain XL1-Blue (Agilent Technologies) was used as a host for routine cloning purposes. Ampicillin (Sigma-Aldrich) to a final concentration of 100 μg/ml was used to select recombinant cells.

### Yeast two-hybrid (Y2H) analyses

The Gal4^DBD^Yap8 fusion was used as a bait protein to screen a yeast cDNA library constructed in the pGADT7-Rec vector (Clontech Laboratories, Inc.). Library construction and screening, by mating, were performed according to Matchmaker™ Gold Yeast Two-hybrid System (Clontech Laboratories, Inc.). Diploid cells displaying histidine and adenine prototrophies and β-galactosidase activity were selected for further studies. The plasmids were isolated, amplified in *E. coli* and re-tested under the same conditions. The resulting prey plasmids were sequenced and the respective DNA sequences identified using the BLAST algorithm.

### Co-immunoprecipitation assays

Co-immunoprecipitation assays were performed as previously described (Soutourina et al., 2006), with minor modifications. Briefly, Y187 cells co-expressing Gal4^DBD^Yap8/Gal4^AD^Ufd2 were grown until early exponential phase (OD_600_ 0.5±0.05) and challenged with 2 mM As(V) for 60 min. Protein extracts were generated in lysis buffer (20 mM Tris-HCl, 150 mM NaCl, 0.5% NP-40, 2 mM EDTA, 10% glycerol) containing a protease inhibitor cocktail (Roche, Mannheim, Germany) and phenylmethylsulfonyl fluoride (PMSF) (Sigma-Aldrich) to a final concentration of 1 mM, by cell disruption with a FastPrep^®^-24 instrument (MP Biomedicals, France). *C*-myc-tagged proteins were immunoprecipitated by the incubation of cell lysates with anti-*c-*myc mouse monoclonal antibody (9E10; Roche; Cat. No. 11 667 149 001), prebound to Dynabeads Pan Mouse IgG (Invitrogen™ Thermo Fisher Scientific Inc.) in a rotating wheel, overnight at 4°C. Immunoprecipitates were washed three times with phosphate-buffered saline (PBS) and were eluted from beads by heating the samples for 10 min at 65°C using Laemmli buffer (62.5 mM Tris-HCl pH 6.8, 2% SDS, 5% β-mercaptoethanol, 10% glycerol, and 0.01% bromophenol blue dye). Immunoprecipitated proteins, along with the whole cell extracts, were loaded on a 12% polyacrylamide gel and were analysed by immunoblotting, as described below, using anti-*c*-myc, anti-HA and anti-Pgk1 antibodies. Reciprocal co-immunoprecipitation assays were performed using BY4742 cells, co-expressing Yap8-*c*-myc/Ufd2-HA, challenged with 1.5 mM As(III) for 90 min. HA-tagged proteins were immunoprecipitated by the incubation of cell lysates with anti-HA mouse monoclonal antibody (12CA5; Roche; Cat. No. 11 583 816 001).

### Cycloheximide chase and immunoblot analysis

Immunoblottings were performed using early exponential phase cells (OD_600_ 0.5±0.05) challenged with 1.5 mM As(III) or 2 mM As(V), and harvested at the indicated time-points. For Yap8-HA stability assays, cells were pre-exposed either to 1.5 mM As(III) or to 2 mM As(V), and washed three times with phosphate-buffered saline (PBS) to remove arsenic compounds. Cells were then resuspended in fresh media supplemented with cycloheximide (CHX) to a final concentration of 0.1 mg/ml, and samples were collected at the indicated time-points. Also, co-treatments with As(III) and CHX were carried out. Total proteins were extracted by the TCA lysis method and protein concentrations were determined using the Bradford protein assay kit (Bio-Rad Laboratories) being then 70–100 µg resolved by SDS-PAGE and transferred to a polyvinylidene fluoride (PVDF) membranes (Millipore, Billerica, MA, USA), using a Trans-Blot Semy Dry transfer system (Bio-Rad Laboratories). Immunoblottings were performed following standard procedures (Ferreira et al., 2012) and using the following antibodies: anti-HA-Peroxidase high affinity rat monoclonal antibody (3F10; Roche; Cat. No. 12 013 819 001), anti-c-myc mouse monoclonal antibody (9E10; Roche; Cat. No. 11 667 149 001), anti-Pgk1 (Invitrogen; Cat. No. 459250), and goat anti-mouse IgG-HRP antibody (Santa Cruz Biotechnology, Dallas, TX, USA; Cat. No. sc-2314). Peroxidase signals were detected using the Super Signal® West Pico and West Femto Maximum Sensitivity Substrates (Thermo Fisher Scientific). Quantification of protein signals was carried out using the ImageJ software (NIH, Bethesda, MD). Pgk1 was used as loading control in all assays. In CHX chase assays, the percentage of proteins present after CHX addition is shown. Protein half-life was estimated as previously described (Boban et al., 2014).

### β-galactosidase assays

β-galactosidase measurements were performed as previously described (Menezes et al., 2004). Briefly, early exponential phase cells subjected or not to Na_2_HAsO_4_ were harvested after 60 min and enzyme activity was assayed by following the degradation of the colorimetric substrate ONPG (o-nitrophenyl-β-D-galactopyraniside) (Sigma-Aldrich) at A__420__ using a microplate spectrophotometer (Epoch™ BioTek). Values were normalized against the number of cells of each culture. Miller units were calculated as previously described (Pimentel et al., 2014). The results are the average of at least three biological replicates (*n*=3).

### Quantitative real-time PCR analyses

RNA was isolated from early exponential phase cultures (OD_600_ 0.5±0.05) that were either non-exposed or exposed to 1.5 mM NaAsO_2_, and harvested at the indicated time-points. RNA samples were treated with the TURBO DNA-free™ kit (Ambion, Cambridge, UK) according to the manufacturer's instructions, and purified by on-column DNAse I digestion using the RNase-Free DNase Set (Qiagen, Hilden, Germany). Total RNA (1 μg) was reverse transcribed with Transcriptor Reverse Transcriptase (Roche). qRT-PCR reactions were performed in the Light Cycler 480 Real-Time PCR System using Light Cycler Fast Start DNA Master SYBR Green I (Roche). Relative standard curves were constructed for each gene, using triplicate serial dilutions of cDNA. The relative expression of the genes was calculated by the relative quantification method with efficiency correction, using the LightCycler Software 4.1 (Roche). Actin (*ACT1*) was used as a reference gene. All assays were made using biological triplicates. The oligonucleotides used are listed in supplementary material Table S3 (oligonucleotides 14 to 23).

### Statistical analysis

The results reported in this study are the averages of at least three independent experiments and are represented as the means±s.d. Differences amongst treatments were detected by the parametric Student's *t*-test using the XLSTAT statistical software 2015.1. Statistical differences between treatments are denoted as *P<0.05 and ***P<0.001.

## Supplementary Material

Supplementary information

## References

[BIO010405C1] Amaral C., Pimentel C., Matos R. G., Arraiano C. M., Matzapetakis M. and Rodrigues-Pousada C. (2013). Two residues in the basic region of the yeast transcription factor Yap8 are crucial for its DNA-binding specificity. *PLoS ONE* 8, e83328 10.1371/journal.pone.0083328 24358276 PMC3865217

[BIO010405C2] Aravind L. and Koonin E. V. (2000). The U box is a modified RING finger — a common domain in ubiquitination. *Curr. Biol.* 10, R132-R134. 10.1016/S0960-9822(00)00398-5 10704423

[BIO010405C3] Batista-Nascimento L., Toledano M. B., Thiele D. J. and Rodrigues-Pousada C. (2013). Yeast protective response to arsenate involves the repression of the high affinity iron uptake system. *Biochim. Biophys. Acta* 1833, 997-1005. 10.1016/j.bbamcr.2012.12.018 23295455

[BIO010405C4] Boban M., Pantazopoulou M., Schick A., Ljungdahl P. O. and Foisner R. (2014). A nuclear ubiquitin-proteasome pathway targets the inner nuclear membrane protein Asi2 for degradation. *J. Cell Sci.* 127, 3603-3613. 10.1242/jcs.153163 24928896 PMC4333764

[BIO010405C5] Bobrowicz P. and Ulaszewski S. (1998). Arsenical-induced transcriptional activation of the yeast *Saccharomyces cerevisiae ACR2* and *ACR3* genes requires the presence of the *ACR1* gene product. *Cell. Mol. Biol. Lett.* 3, 13-20.

[BIO010405C6] Bohm S., Lamberti G., Fernandez-Saiz V., Stapf C. and Buchberger A. (2011). Cellular functions of Ufd2 and Ufd3 in proteasomal protein degradation depend on Cdc48 binding. *Mol. Cell. Biol.* 31, 1528-1539. 10.1128/MCB.00962-10 21282470 PMC3135295

[BIO010405C7] Chatterjee A., Upadhyay S., Chang X., Nagpal J. K., Trink B. and Sidransky D. (2008). U-box-type ubiquitin E4 ligase, UFD2a attenuates cisplatin mediated degradation of DeltaNp63alpha. *Cell Cycle* 7, 1231-1237. 10.4161/cc.7.9.5795 18418053 PMC3073353

[BIO010405C8] Chen L. and Madura K. (2002). Rad23 promotes the targeting of proteolytic substrates to the proteasome. *Mol. Cell. Biol.* 22, 4902-4913. 10.1128/MCB.22.13.4902-4913.2002 12052895 PMC133919

[BIO010405C9] Ciechanover A. and Stanhill A. (2014). The complexity of recognition of ubiquitinated substrates by the 26S proteasome. *Biochim. Biophys. Acta.* 1843, 86-96. 10.1016/j.bbamcr.2013.07.007 23872423

[BIO010405C10] Di Y. and Tamas M. J. (2007). Regulation of the arsenic-responsive transcription factor Yap8p involves the ubiquitin-proteasome pathway. *J. Cell Sci.* 120, 256-264. 10.1242/jcs.03346 17200139

[BIO010405C11] Ferreira R. T., Silva A. R. C., Pimentel C., Batista-Nascimento L., Rodrigues-Pousada C. and Menezes R. A. (2012). Arsenic stress elicits cytosolic Ca(2+) bursts and Crz1 activation in *Saccharomyces cerevisiae*. *Microbiology* 158, 2293-2302. 10.1099/mic.0.059170-0 22745270

[BIO010405C12] Finley D. (2009). Recognition and processing of ubiquitin-protein conjugates by the proteasome. *Annu. Rev. Biochem.* 78, 477-513. 10.1146/annurev.biochem.78.081507.101607 19489727 PMC3431160

[BIO010405C13] Flora S. J. S. (2011). Arsenic-induced oxidative stress and its reversibility. *Free Radic. Biol. Med.* 51, 257-281. 10.1016/j.freeradbiomed.2011.04.008 21554949

[BIO010405C14] Funakoshi M., Sasaki T., Nishimoto T. and Kobayashi H. (2002). Budding yeast Dsk2p is a polyubiquitin-binding protein that can interact with the proteasome. *Proc. Natl. Acad. Sci. USA* 99, 745-750. 10.1073/pnas.012585199 11805328 PMC117376

[BIO010405C15] Gueldener U., Heinisch J., Koehler G. J., Voss D. and Hegemann J. H. (2002). A second set of loxP marker cassettes for Cre-mediated multiple gene knockouts in budding yeast. *Nucleic Acids Res.* 30, e23 10.1093/nar/30.6.e23 11884642 PMC101367

[BIO010405C16] Hatakeyama S. and Nakayama K.-I. I. (2003). U-box proteins as a new family of ubiquitin ligases. *Biochem. Biophys. Res. Commun.* 302, 635-645. 10.1016/S0006-291X(03)00245-6 12646216

[BIO010405C17] Haugen A. C., Kelley R., Collins J. B., Tucker C. J., Deng C., Afshari C. A., Brown J. M., Ideker T. and Van Houten B. (2004). Integrating phenotypic and expression profiles to map arsenic-response networks. *Genome Biol.* 5, R95 10.1186/gb-2004-5-12-r95 15575969 PMC545798

[BIO010405C18] Hochstrasser M. (2009). Introduction to intracellular protein degradation. *Chem. Rev.* 109, 1479-1480. 10.1021/cr900054t 19253968

[BIO010405C19] Hoppe T. (2005). Multiubiquitylation by E4 enzymes: ‘one size’ doesn't fit all. *Trends Biochem. Sci.* 30, 183-187. 10.1016/j.tibs.2005.02.004 15817394

[BIO010405C20] Hosoda M., Ozaki T., Miyazaki K., Hayashi S., Furuya K., Watanabe K.-I., Nakagawa T., Hanamoto T., Todo S. and Nakagawara A. (2005). UFD2a mediates the proteasomal turnover of p73 without promoting p73 ubiquitination. *Oncogene* 24, 7156-7169. 10.1038/sj.onc.1208872 16170377

[BIO010405C21] Iland H. J. and Seymour J. F. (2013). Role of arsenic trioxide in acute promyelocytic leukemia. *Curr. Treat. Options Oncol.* 14, 170-184. 10.1007/s11864-012-0223-3 23322117

[BIO010405C22] Ilina Y., Sloma E., Maciaszczyk-Dziubinska E., Novotny M., Thorsen M., Wysocki R. and Tamas M. J. (2008). Characterization of the DNA-binding motif of the arsenic-responsive transcription factor Yap8p. *Biochem. J.* 415, 467-475. 10.1042/BJ20080713 18593383

[BIO010405C23] Jacobson T., Navarrete C., Sharma S. K., Sideri T. C., Ibstedt S., Priya S., Grant C. M., Christen P., Goloubinoff P. and Tamas M. J. (2012). Arsenite interferes with protein folding and triggers formation of protein aggregates in yeast. *J. Cell Sci.* 125, 5073-5083. 10.1242/jcs.107029 22946053

[BIO010405C24] Jentsch S., Seufert W., Sommer T. and Reins H.-A. (1990). Ubiquitin-conjugating enzymes: novel regulators of eukaryotic cells. *Trends Biochem. Sci.* 15, 195-198. 10.1016/0968-0004(90)90161-4 2193438

[BIO010405C25] Jomova K., Jenisova Z., Feszterova M., Baros S., Liska J., Hudecova D., Rhodes C. J. and Valko M. (2011). Arsenic: toxicity, oxidative stress and human disease. *J. Appl. Toxicol.* 31, 95-107. 10.1002/jat.1649 21321970

[BIO010405C26] Kim I., Mi K. and Rao H. (2004). Multiple interactions of rad23 suggest a mechanism for ubiquitylated substrate delivery important in proteolysis. *Mol. Biol. Cell* 15, 3357-3365. 10.1091/mbc.E03-11-0835 15121879 PMC452589

[BIO010405C27] Koegl M., Hoppe T., Schlenker S., Ulrich H. D., Mayer T. U. and Jentsch S. (1999). A novel ubiquitination factor, E4, is involved in multiubiquitin chain assembly. *Cell* 96, 635-644. 10.1016/S0092-8674(00)80574-7 10089879

[BIO010405C28] Li B. and Fields S. (1993). Identification of mutations in p53 that affect its binding to SV40 large T antigen by using the yeast two-hybrid system. *FASEB J.* 7, 957-963. 8344494 10.1096/fasebj.7.10.8344494

[BIO010405C29] Liu C., van Dyk D., Xu P., Choe V., Pan H., Peng J., Andrews B. and Rao H. (2010). Ubiquitin chain elongation enzyme Ufd2 regulates a subset of Doa10 substrates. *J. Biol. Chem.* 285, 10265-10272. 10.1074/jbc.M110.110551 20159987 PMC2856231

[BIO010405C30] Liu C., van Dyk D., Choe V., Yan J., Majumder S., Costanzo M., Bao X., Boone C., Huo K., Winey M. et al. (2011). Ubiquitin ligase Ufd2 is required for efficient degradation of Mps1 kinase. *J. Biol. Chem.* 286, 43660-43667. 10.1074/jbc.M111.286229 22045814 PMC3243506

[BIO010405C31] Mandal B. K. and Suzuki K. T. (2002). Arsenic round the world: a review. *Talanta* 58, 201-235. 10.1016/S0039-9140(02)00268-0 18968746

[BIO010405C32] Marek A. and Korona R. (2013). Restricted pleiotropy facilitates mutational erosion of major life-history traits. *Evolution* 67, 3077-3086. 10.1111/evo.12196 24151994

[BIO010405C33] Mathews V., Chendamarai E., George B., Viswabandya A. and Srivastava A. (2011). Treatment of acute promyelocytic leukemia with single-agent arsenic trioxide. *Mediterr. J. Hematol. Infect. Dis.* 3, e2011056 10.4084/mjhid.2011.056 22220253 PMC3248333

[BIO010405C34] Menezes R. A., Amaral C., Delaunay A., Toledano M. and Rodrigues-Pousada C. (2004). Yap8p activation in *Saccharomyces cerevisia*e under arsenic conditions. *FEBS Lett.* 566, 141-146. 10.1016/j.febslet.2004.04.019 15147884

[BIO010405C35] Menezes R. A., Amaral C., Batista-Nascimento L., Santos C., Ferreira R. B., Devaux F., Eleutherio E. C. A. and Rodrigues-Pousada C. (2008). Contribution of Yap1 towards *Saccharomyces cerevisiae* adaptation to arsenic-mediated oxidative stress. *Biochem. J.* 414, 301-311. 10.1042/BJ20071537 18439143

[BIO010405C36] Migdal I., Ilina Y., Tamas M. J. and Wysocki R. (2008). Mitogen-activated protein kinase Hog1 mediates adaptation to G1 checkpoint arrest during arsenite and hyperosmotic stress. *Eukaryot. Cell* 7, 1309-1317. 10.1128/EC.00038-08 18552285 PMC2519783

[BIO010405C37] Moll U. M. and Petrenko O. (2003). The MDM2-p53 interaction. *Mol. Cancer Res.* 1, 1001-1008. 14707283

[BIO010405C38] Ongkeko W. M., Wang X. Q., Siu W. Y., Lau A. W. S., Yamashita K., Harris A. L., Cox L. S. and Poon R. Y. C. (1999). MDM2 and MDMX bind and stabilize the p53-related protein p73. *Curr. Biol.* 9, 829-832. 10.1016/S0960-9822(99)80367-4 10469568

[BIO010405C39] Pimentel C., Caetano S. M., Menezes R., Figueira I., Santos C. N., Ferreira R. B., Santos M. A. S. and Rodrigues-Pousada C. (2014). Yap1 mediates tolerance to cobalt toxicity in the yeast *Saccharomyces cerevisiae*. *Biochim. Biophys. Acta* 1840, 1977-1986. 10.1016/j.bbagen.2014.01.032 24486411

[BIO010405C40] Ratnaike R. N. (2003). Acute and chronic arsenic toxicity. *Postgrad. Med. J.* 79, 391-396. 10.1136/pmj.79.933.391 12897217 PMC1742758

[BIO010405C41] Rodrigues-Pousada C. A., Nevitt T., Menezes R., Azevedo D., Pereira J. and Amaral C. (2004). Yeast activator proteins and stress response: an overview. *FEBS Lett.* 567, 80-85. 10.1016/j.febslet.2004.03.119 15165897

[BIO010405C42] Sommer T. and Seufert W. (1992). Genetic analysis of ubiquitin-dependent protein degradation. *Experientia* 48, 172-178. 10.1007/BF01923510 1740189

[BIO010405C43] Soutourina J., Bordas-Le Floch V., Gendrel G., Flores A., Ducrot C., Dumay-Odelot H., Soularue P., Navarro F., Cairns B. R., Lefebvre O. et al. (2006). Rsc4 connects the chromatin remodeler RSC to RNA polymerases. *Mol. Cell. Biol.* 26, 4920-4933. 10.1128/MCB.00415-06 16782880 PMC1489167

[BIO010405C44] Thorsen M., Jacobson T., Vooijs R., Navarrete C., Bliek T., Schat H. and Tamás M. J. (2012). Glutathione serves an extracellular defence function to decrease arsenite accumulation and toxicity in yeast. *Mol. Microbiol.* 84, 1177-1188. 10.1111/j.1365-2958.2012.08085.x 22554109

[BIO010405C45] Tu D., Li W., Ye Y. and Brunger A. T. (2007). Structure and function of the yeast U-box-containing ubiquitin ligase Ufd2p. *Proc. Natl. Acad. Sci. USA* 104, 15599-15606. 10.1073/pnas.0701369104 17890322 PMC2000413

[BIO010405C46] Wysocki R., Bobrowicz P. and Ulaszewski S. (1997). The *Saccharomyces cerevisiae ACR3* gene encodes a putative membrane protein involved in arsenite transport. *J. Biol. Chem.* 272, 30061-30066. 10.1074/jbc.272.48.30061 9374482

[BIO010405C47] Wysocki R., Fortier P.-K., Maciaszczyk E., Thorsen M., Leduc A., Odhagen A., Owsianik G., Ulaszewski S., Ramotar D. and Tamas M. J. (2004). Transcriptional activation of metalloid tolerance genes in *Saccharomyces cerevisiae* requires the AP-1-like proteins Yap1p and Yap8p. *Mol. Biol. Cell* 15, 2049-2060. 10.1091/mbc.E03-04-0236 14978214 PMC404003

[BIO010405C48] Yoshikawa K., Tanaka T., Ida Y., Furusawa C., Hirasawa T. and Shimizu H. (2011). Comprehensive phenotypic analysis of single-gene deletion and overexpression strains of *Saccharomyces cerevisiae*. *Yeast* 28, 349-361. 10.1002/yea.1843 21341307

