# Supplementary material for: E4-Ubiquitin ligase Ufd2 stabilizes Yap8 and modulates arsenic stress responses independent of the U-box motif
Source: Biol Open. 2015 Aug 14;4(9):1122–31. doi: 10.1242/bio.010405 (PMC4582114; doi:10.1242/bio.010405)
Supplement: Supplementary information [file supp_4_9_1122__index.html]

E4-Ubiquitin ligase Ufd2 stabilizes Yap8 and modulates arsenic stress responses independent of the U-box motif — Supplementary information 

# E4-Ubiquitin ligase Ufd2 stabilizes Yap8 and modulates arsenic stress responses independent of the U-box motif

## BIO010405 Supplementary information

**Files in this Data Supplement:**

- Supplementary information
