# Supplementary material for: E4-Ubiquitin ligase Ufd2 stabilizes Yap8 and modulates arsenic stress responses independent of the U-box motif
Source: Biol Open. 2015 Aug 14;4(9):1122–31. doi: 10.1242/bio.010405 (PMC4582114; doi:10.1242/bio.010405)
Supplement: Supplementary information [file supp_bio.010405_BIO010405supp.pdf]

Supplementary Material

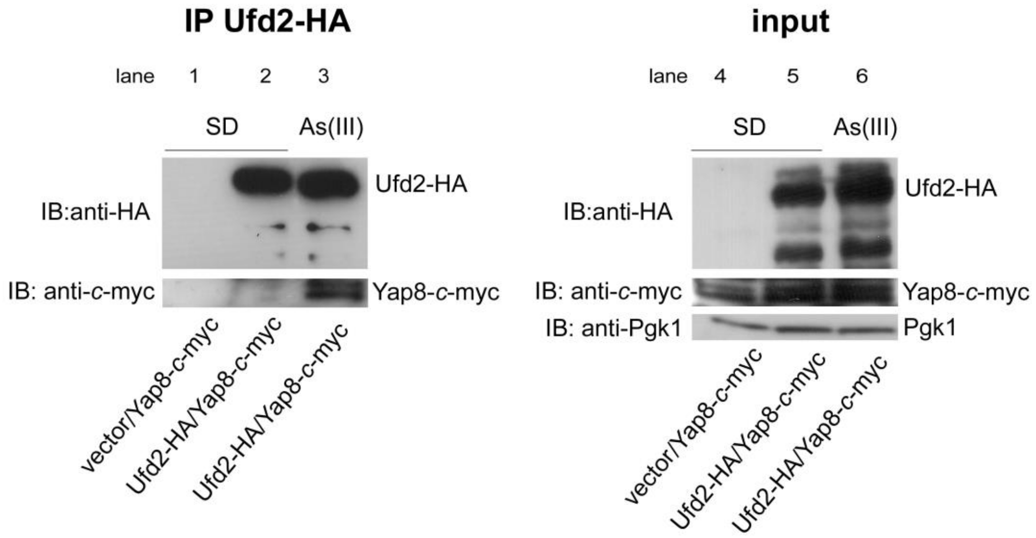


**Fig. S1. Reciprocal co-immunoprecipitation assays showing arsenic-mediated interaction between Yap8 and Ufd2.** BY4742 cells co-transformed with vector/*YAP8*-*c*-myc (lanes 1 and 4) or *UFD2*-HA/*YAP8*-*c*-myc (lanes 2, 3, 5 and 6) were exposed or not to 1.5 mM As(III) for 90 min and Ufd2-HA was immunoprecipitated with anti-HA antibody. Immunoblotting was performed using anti-HA, anti-*c*-myc and anti-Pgk1 antibodies. A representative experiment is shown. IP – immunoprecipitation; IB – immunoblotting; SD – Synthetic Defined medium, control condition.

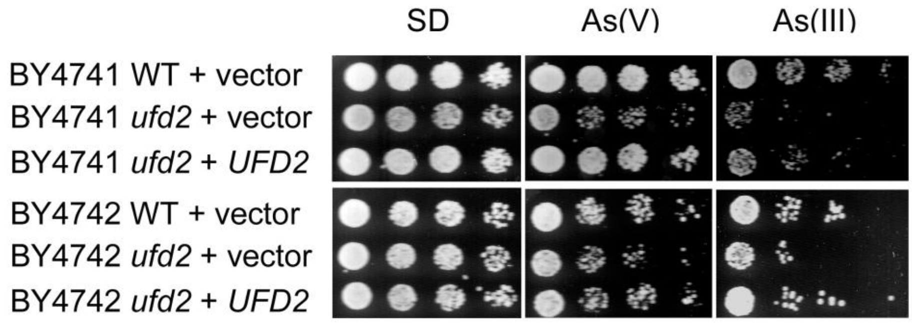


**Fig. S2. As(V)- and As(III)-sensitivity phenotypes of *ufd2* are restored by expressing an episomal copy of *UFD2*.** Exponential phase BY4741 and BY4742 wild type (WT) and *ufd2* cells expressing an episomal copy of *UFD2* or the respective control vector were serially diluted and spotted onto SD media supplemented or not with 2 mM As(V) or 1.5 mM As(III). Growth was recorded after 2 days incubation at 30ºC. A representative experiment is shown.

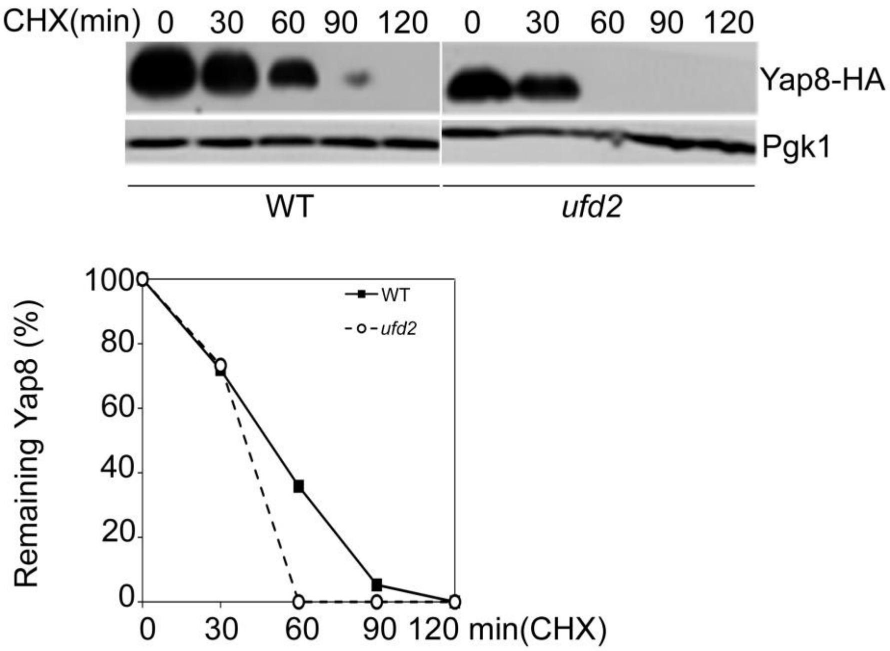


**Fig. S3. Ufd2 mediates Yap8 stabilization under As(V) stress conditions.** BY4742 wild type (WT) and *ufd2* mutant strains expressing Yap8-HA were pre-treated with 2 mM As(V) for 60 min, washed and subsequently treated with 0.1 mg/mL cycloheximide (CHX) up to 120 min prior to immunoblotting using anti-HA and anti-Pgk1 antibodies. The graph represents the percentage of remaining Yap8 protein after CHX addition. A representative experiment is shown.

**Table S1.** *Saccharomyces cerevisiae* strains used in this study

| Strain | Genotype | Reference or source |
|---|---|---|
| BY4742 | *MATα his3Δ1 leu2Δ0 lys2Δ0* ura3Δ0 | EUROSCARF* |
| *ufd2* | *MATα his3Δ1 leu2Δ0* lys2Δ0 *ura3Δ0 YDL190c::kanMX4* | EUROSCARF |
| *yap8* | *MATα his3Δ1 leu2Δ0* lys2Δ0 *ura3Δ0 YPR199c::kanMX4* | EUROSCARF |
| *yap8ufd2* | *MATα his3Δ1 leu2Δ0* lys2Δ0 *ura3Δ0 YPR199c::kanMX4 YDL190c::HIS3MX4* | This study |
| *ubc4* | *MATα his3Δ1 leu2Δ0 lys2Δ0 ura3Δ0 YBR082c::kanMX4* | ATCC** |
| *rad23* | *MATα his3Δ1 leu2Δ0 lys2Δ0 ura3Δ0 YEL037c::kanMX4* | EUROSCARF |
| *dsk2* | *MATα his3Δ1 leu2Δ0 lys2Δ0 ura3Δ0 YMR276w::kanMX4* | EUROSCARF |
| Y187 | *MATα ura3-52 his3-200 ade2-101 trp1-901 leu2-3 112 gal4Δ met-gal80Δ URA3::GAL1UAS-GAL1TATA-lacZ MEL1* | Clontech Laboratories, Inc. |
| BY4741 | *MAT*a *his3Δ1 leu2Δ0 met15Δ0* ura3Δ0 | EUROSCARF |
| *ufd2* | *MATa his3Δ1 leu2Δ0 met15Δ0 ura3Δ0 YDL190C::kanMX4* | EUROSCARF |
| $Ufd2^{U\text{-}box\Delta}$ | *MATa his3Δ1 leu2Δ0 met15Δ0 ura3Δ0* $YDL190C^{2568\text{-}2886\ bp}$*::HIS3* | Chang Liu *et al.* (2011) |

*EUROpean Saccharomyces Cerevisiae ARchive for Functional analysis

** American Type Culture Collection

**Table S2.** Plasmids used in this study

| Name | Features | Reference or source |
|---|---|---|
| pRS416 | *CEN*, *URA3* | Agilent Technologies |
| pRS416-*YAP8-HA* | | Our unpublished work |
| pRS416-*YAP8-c-myc* | | Amaral et al. (2013) |
| pRS416-*UFD2-HA* | | This study |
| pGADT7-Rec | 2µ, *LEU2, HA* | Clontech |
| pGADT7-*T-antigen* | | Clontech |
| pGADT7-*UFD2* | | This study |
| pGBKT7 | 2µ, *TRP1*, *c-myc* | Clontech |
| pGBKT7-*LamC* | | Clontech |
| pGBKT7-*p53* | | Clontech |
| pGBKT7-*YAP8* | | Our unpublished work |
| YCplac111 | *CEN*, *LEU2* | Agilent Technologies |
| YCplac111-*UFD2-HA* | | This study |
| YEplac181 | 2µ, *LEU2* | Agilent Technologies |
| YEplac181-*YAP8-HA* | | Our unpublished work |
| pGal-*MPS1-c-myc* | *Integrative, URA3, GAL1$^{promoter}$* | Chang Liu et al. (2011) |

Biology Open • Supplementary information

**Table S3.** Oligonucleotides used in this study

| # | Name | Sequence (5' – 3' UTR) |
|---|---|---|
| 1 | UFD2-HIS-Fw [D] | GGGAAAAGTTAACTTTGAAAGTAGAACCCTCATTCCATAGATCGTAC GCTGCAGG |
| 2 | UFD2-HIS-Rv [D] | TTGATTAGGGTCAATTTTGCAATTTATTCTATCACTTATTTTAGGGAG ACCGGCAGAT |
| 3 | A1-UFD2[D] | GGCTTGCTGGTACAATATGG |
| 4 | A4-UFD2[D] | CCAGAGCTTTGAGAAGAG |
| 5 | UFD2 400-Fw* | GAAAGGTAAAGTTGAC |
| 6 | UFD2 1300-Fw* | CTTAAACTCAAGGAC |
| 7 | UFD2 2200-Fw* | GGTAAATTAGTGCAG |
| 8 | UFD2-pGADT7-Fw[C] | ACCGCCATAGAAGATATTTTAC |
| 9 | UFD2-pGADT7-Rv[C] | TCACTCGCTTGCTTTATG |
| 10 | pRS416-*SmaI*-UFD2-P1000-Fw [C] | GAATTCCTGCAGCCCAATATTCTGTTATTG |
| 11 | HA-UFD2-Rv[C] | AGCGTAATCTGGAACATCGTATGGGTACATTCACTTATTCATTCA |
| 12 | HA-UFD2-Term500-Fw[C] | ATGTACCCATACGATGTTCCAGATTACGCTTAGAATAAATTGCAA |
| 13 | UFD2-Term500-pRS416-*SmaI*-Rv[C] | ACTAGTGGATCCCCCAGTTGGCTGAATTGA |
| 14 | UFD2-Fw● | GACTTTCCTGTTGGATGAAG |
| 15 | UFD2-Rv● | CTCTTCCTCTCTTGTTGGTG |
| 16 | ACR2-Fw● | AGGCAACTCAAGGCCTAAT |
| 17 | ACR2-Rv● | GAACATGCCAAGCGTTTGTA |
| 18 | ACR3-Fw● | AAGAGGGTCTGGGGAAGAAA |
| 19 | ACR3-Rv● | GCAATTGCCAGGGATAGTTC |
| 20 | YAP8-Fw● | AACCGCCCACATGTAACACT |
| 21 | YAP8-Rv● | TCCAACACACACTGAGAGCAG |
| 22 | ACT1-Fw● | CTATTGGTAACGAAAGATTCA |
| 23 | ACT1-Rv● | CCTTACGGACATCGACATCA |

Fw - forward; Rv - reverse

[D]Gene disruption and mutant confirmation, *Sequencing, [C] Cloning, ●qRT-PCR

Biology Open • Supplementary information
